# Ethyl 6-[4-(dimethyl­amino)phen­yl]-4-hydr­oxy-2-oxo-4-(trifluoro­methyl)­hexa­hydro­pyrimidine-5-carboxyl­ate

**DOI:** 10.1107/S1600536810013127

**Published:** 2010-04-14

**Authors:** Xiao-Ping Song, Gong-Chun Li, Chang-Zeng Wu, Feng-Ling Yang

**Affiliations:** a College of Chemistry and Chemical Engineering, Xuchang University, Henan Province 461000, People’s Republic of China

## Abstract

The title compound, C_16_H_20_F_3_N_3_O_4_, was prepared by reaction of 4-(dimethyl­amino)benzaldehyde, ethyl 4,4,4-trifluoro-3-oxo­butanoate and urea. In the title mol­ecule, the pyrimidine ring adopts a half-chair conformation and there is an intra­molecular hydrogen bond (O—H⋯O). The crystal structure is stabilized by two types inter­molecular hydrogen bonds (N—H⋯O and N—H⋯N).

## Related literature

For the bioactivity of dihydro­pyrimidines, see: Brier *et al.* (2004 ▶); Cochran *et al.* (2005 ▶); Moran *et al.* (2007 ▶); Zorkun *et al.* (2006 ▶). For the bioactivity of organofluorine compounds, see: Hermann *et al.* (2003 ▶); Ulrich (2004 ▶).
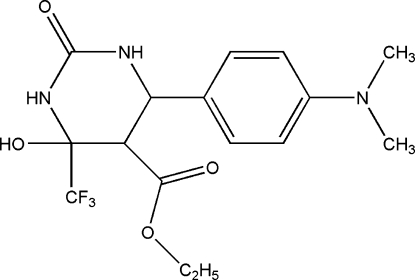


## Experimental

### 

#### Crystal data

C_16_H_20_F_3_N_3_O_4_
*M* *_r_* = 375.35
Monoclinic, 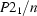

*a* = 13.319 (4) Å
*b* = 7.923 (2) Å
*c* = 16.530 (5) Å
β = 92.720 (5)°
*V* = 1742.3 (9) Å^3^
*Z* = 4
Mo *K*α radiation
μ = 0.12 mm^−1^
*T* = 116 K
0.24 × 0.20 × 0.14 mm

#### Data collection

Rigaku Saturn CCD area-detector diffractometer
Absorption correction: multi-scan (*CrystalClear*; Rigaku/MSC, 2005 ▶) *T*_min_ = 0.971, *T*_max_ = 0.983
11487 measured reflections
3081 independent reflections
2522 reflections with *I* > 2σ(*I*)
*R*_int_ = 0.030

#### Refinement

*R*[*F* ^2^ > 2σ(*F* ^2^)] = 0.037
*wR*(*F* ^2^) = 0.107
*S* = 1.07
3081 reflections
250 parameters
3 restraints
H atoms treated by a mixture of independent and constrained refinement
Δρ_max_ = 0.17 e Å^−3^
Δρ_min_ = −0.24 e Å^−3^

### 

Data collection: *CrystalClear* (Rigaku/MSC, 2005 ▶); cell refinement: *CrystalClear*; data reduction: *CrystalClear*; program(s) used to solve structure: *SHELXS97* (Sheldrick, 2008 ▶); program(s) used to refine structure: *SHELXL97* (Sheldrick, 2008 ▶); molecular graphics: *SHELXTL* (Sheldrick, 2008 ▶); software used to prepare material for publication: *CrystalStructure* (Rigaku/MSC, 2005 ▶).

## Supplementary Material

Crystal structure: contains datablocks global, I. DOI: 10.1107/S1600536810013127/om2331sup1.cif

Structure factors: contains datablocks I. DOI: 10.1107/S1600536810013127/om2331Isup2.hkl

Additional supplementary materials:  crystallographic information; 3D view; checkCIF report

## Figures and Tables

**Table 1:** Hydrogen-bond geometry (Å, °)

*D*—H⋯*A*	*D*—H	H⋯*A*	*D*⋯*A*	*D*—H⋯*A*
O1—H1⋯O3	0.86 (1)	1.97 (1)	2.7601 (16)	153 (2)
N1—H1*A*⋯O2^i^	0.90 (1)	1.91 (1)	2.8049 (15)	174 (2)
N2—H2*A*⋯N3^ii^	0.90 (1)	2.12 (1)	3.0241 (18)	178 (2)

Symmetry codes: (i) 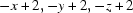; (ii) 
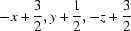
.

**Fig. 1.**
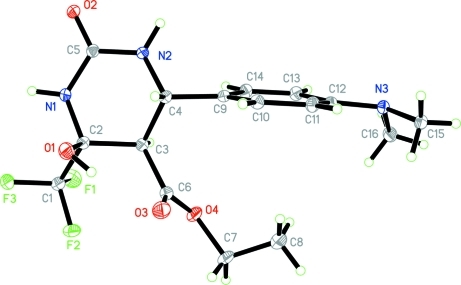
The asymmetric unit of the title compound, (I), with displacement ellipsoids drawn at the 30% probability level.

**Fig. 2.**
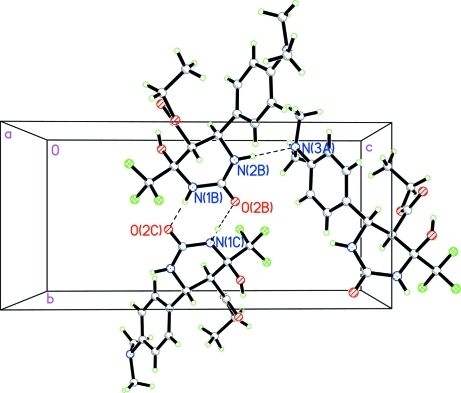
The packing diagram of the title compound. Intermolecular hydrogen bonds are shown as dashed line.

## supplementary crystallographic information

### Comment

Dihydropyrimidine (DHPM) derivatives can be used as potential calcium channel blockers (Zorkun *et al.*, 2006), inhibitors of mitotic kinesin Eg5 for treating cancer (Cochran *et al.*, 2005; Brier *et al.*, 2004) and as TRPA1 modulators for treating pain (Moran *et al.*, 2007). Besides, compounds that contain fluorine have special bioactivity, for example, flumioxazin is a widely used herbicide (Hermann *et al.*, 2003; Ulrich, 2004). This led us to pay much attention to the synthesis and bioactivity of these important fused perfluoroalkylated heterocyclic compounds. During the synthesis of DHPM derivatives, the title compound, an intermediate, was isolated and confirmed by X-ray diffraction to elucidate the reaction mechanism. We report here the crystal structure of the title compound(Fig. 1).

In the title molecule, the pyrimidine ring adopts a half-chair conformation, and there is an intramolecular hydrogen bond (O—H···O). The crystal structure is stabilized by two types intermolecular hydrogen bonds (N—H···O, and N—H···N).

### Experimental

The title compound was synthesized by 4-(dimethylamino)-benzaldehyde (2.98 g, 20 mmol), ethyl 4,4,4-trifluoro-3-oxobutanoate(4.42 g, 24 mmol), and urea (1.80 g, 30 mmol), catalyzed by sulfamic acid(0.6 g), in the solvent of ethanol(20 ml), by refluxing for 3 h under the conditions of stirring. The solvent was evaporated in vacuo and the residue was washed with water. The title compound was recrystallized from ethanol and single crystals of (I) were obtained by slow evaporation.

### Refinement

H atoms of N and O were positioned freely refined. The other H atoms were placed in calculated positions, with C—H = 0.93, 0.96, 0.97 or 0.98 Å, and included in the final cycles of refinement using a riding model, with *U*_iso_(H) = 1.2Ueq(C).

### Crystal data

**Table:**

C_16_H_20_F_3_N_3_O_4_	*F*(000) = 784
*M**_r_* = 375.35	*D*_x_ = 1.431 Mg m^−^^3^
Monoclinic, *P*2_1_/*n*	Mo *K*α radiation, λ = 0.71073 Å
*a* = 13.319 (4) Å	Cell parameters from 6970 reflections
*b* = 7.923 (2) Å	θ = 1.5–27.9°
*c* = 16.530 (5) Å	µ = 0.12 mm^−^^1^
β = 92.720 (5)°	*T* = 116 K
*V* = 1742.3 (9) Å^3^	Prism, colorless
*Z* = 4	0.24 × 0.20 × 0.14 mm

### Data collection

**Table:**

Rigaku Saturn CCD area-detector diffractometer	3081 independent reflections
Radiation source: rotating anode	2522 reflections with *I* > 2σ(*I*)
multilayer	*R*_int_ = 0.030
Detector resolution: 14.63 pixels mm^-1^	θ_max_ = 25.0°, θ_min_ = 1.9°
ω and φ scans	*h* = −15→15
Absorption correction: multi-scan (*CrystalClear*; Rigaku/MSC, 2005)	*k* = −9→9
*T*_min_ = 0.971, *T*_max_ = 0.983	*l* = −19→18
11487 measured reflections	

### Refinement

**Table:**

Refinement on *F*^2^	Primary atom site location: structure-invariant direct methods
Least-squares matrix: full	Secondary atom site location: difference Fourier map
*R*[*F*^2^ > 2σ(*F*^2^)] = 0.037	Hydrogen site location: inferred from neighbouring sites
*wR*(*F*^2^) = 0.107	H atoms treated by a mixture of independent and constrained refinement
*S* = 1.07	*w* = 1/[σ^2^(*F*_o_^2^) + (0.0725*P*)^2^] where *P* = (*F*_o_^2^ + 2*F*_c_^2^)/3
3081 reflections	(Δ/σ)_max_ = 0.001
250 parameters	Δρ_max_ = 0.17 e Å^−^^3^
3 restraints	Δρ_min_ = −0.24 e Å^−^^3^

### Special details

**Table:**

Geometry. All esds (except the esd in the dihedral angle between two l.s. planes) are estimated using the full covariance matrix. The cell esds are taken into account individually in the estimation of esds in distances, angles and torsion angles; correlations between esds in cell parameters are only used when they are defined by crystal symmetry. An approximate (isotropic) treatment of cell esds is used for estimating esds involving l.s. planes.
Refinement. Refinement of *F*^2^ against ALL reflections. The weighted *R*-factor wR and goodness of fit *S* are based on *F*^2^, conventional *R*-factors *R* are based on *F*, with *F* set to zero for negative *F*^2^. The threshold expression of *F*^2^ > σ(*F*^2^) is used only for calculating *R*-factors(gt) etc. and is not relevant to the choice of reflections for refinement. *R*-factors based on *F*^2^ are statistically about twice as large as those based on *F*, and *R*- factors based on ALL data will be even larger.

### Fractional atomic coordinates and isotropic or equivalent isotropic displacement parameters (Å^2^)

**Table:**

	*x*	*y*	*z*	*U*_iso_*/*U*_eq_	
F2	0.76872 (7)	0.70609 (12)	1.19210 (5)	0.0422 (3)	
F3	0.87687 (7)	0.90599 (12)	1.18384 (5)	0.0419 (3)	
F1	0.73912 (7)	0.91322 (12)	1.11033 (6)	0.0434 (3)	
O1	0.93596 (8)	0.60732 (13)	1.11199 (6)	0.0292 (3)	
O2	0.96896 (7)	0.91625 (12)	0.90354 (6)	0.0262 (3)	
O3	0.78789 (9)	0.36331 (13)	1.10635 (6)	0.0385 (3)	
O4	0.63911 (8)	0.47358 (14)	1.06174 (7)	0.0362 (3)	
N1	0.90896 (9)	0.83310 (15)	1.02318 (7)	0.0231 (3)	
N2	0.87955 (8)	0.67473 (14)	0.90588 (7)	0.0216 (3)	
N3	0.57474 (8)	0.09436 (13)	0.76740 (7)	0.0214 (3)	
C1	0.81144 (11)	0.81114 (19)	1.14029 (9)	0.0296 (4)	
C2	0.86240 (10)	0.71282 (17)	1.07362 (8)	0.0220 (3)	
C3	0.78493 (10)	0.60970 (16)	1.02222 (8)	0.0208 (3)	
H3	0.7317	0.6850	1.0007	0.025*	
C4	0.84102 (10)	0.53309 (17)	0.95139 (8)	0.0198 (3)	
H4	0.8980	0.4669	0.9736	0.024*	
C5	0.92246 (10)	0.81105 (16)	0.94208 (8)	0.0202 (3)	
C6	0.73872 (11)	0.46902 (19)	1.06923 (9)	0.0275 (3)	
C7	0.58484 (14)	0.3318 (2)	1.09539 (11)	0.0478 (5)	
H7A	0.5231	0.3717	1.1176	0.057*	
H7B	0.6256	0.2799	1.1388	0.057*	
C8	0.56152 (16)	0.2067 (2)	1.03137 (11)	0.0555 (6)	
H8A	0.5235	0.2597	0.9876	0.083*	
H8B	0.5229	0.1164	1.0530	0.083*	
H8C	0.6230	0.1625	1.0118	0.083*	
C9	0.77484 (10)	0.41978 (16)	0.89851 (8)	0.0190 (3)	
C10	0.79225 (10)	0.24776 (18)	0.89719 (8)	0.0213 (3)	
H10	0.8479	0.2040	0.9262	0.026*	
C11	0.72844 (10)	0.13904 (17)	0.85356 (8)	0.0219 (3)	
H11	0.7419	0.0239	0.8535	0.026*	
C12	0.64423 (10)	0.20114 (16)	0.80974 (8)	0.0195 (3)	
C13	0.63014 (10)	0.37648 (17)	0.80716 (8)	0.0225 (3)	
H13	0.5772	0.4215	0.7754	0.027*	
C14	0.69393 (10)	0.48298 (17)	0.85123 (8)	0.0226 (3)	
H14	0.6827	0.5988	0.8493	0.027*	
C15	0.58978 (11)	−0.08702 (17)	0.77979 (8)	0.0254 (3)	
H15A	0.6557	−0.1179	0.7640	0.038*	
H15B	0.5403	−0.1483	0.7476	0.038*	
H15C	0.5832	−0.1137	0.8359	0.038*	
C16	0.46919 (10)	0.14840 (19)	0.77106 (9)	0.0301 (4)	
H16A	0.4564	0.1830	0.8253	0.045*	
H16B	0.4256	0.0561	0.7557	0.045*	
H16C	0.4568	0.2412	0.7346	0.045*	
H1	0.9050 (13)	0.5138 (16)	1.1199 (12)	0.054 (6)*	
H1A	0.9442 (11)	0.9186 (15)	1.0458 (9)	0.032 (4)*	
H2A	0.8942 (11)	0.6524 (19)	0.8542 (6)	0.032 (4)*	

### Atomic displacement parameters (Å^2^)

**Table:**

	*U*^11^	*U*^22^	*U*^33^	*U*^12^	*U*^13^	*U*^23^
F2	0.0505 (6)	0.0475 (6)	0.0302 (5)	−0.0197 (5)	0.0198 (4)	−0.0068 (4)
F3	0.0473 (6)	0.0502 (6)	0.0291 (5)	−0.0227 (5)	0.0115 (4)	−0.0174 (4)
F1	0.0429 (6)	0.0419 (6)	0.0464 (6)	0.0076 (4)	0.0123 (5)	−0.0114 (4)
O1	0.0280 (6)	0.0337 (6)	0.0254 (6)	−0.0022 (5)	−0.0052 (4)	0.0032 (5)
O2	0.0299 (6)	0.0269 (5)	0.0219 (5)	−0.0110 (4)	0.0029 (4)	0.0007 (4)
O3	0.0505 (8)	0.0332 (6)	0.0315 (6)	−0.0106 (5)	−0.0021 (5)	0.0088 (5)
O4	0.0305 (7)	0.0399 (7)	0.0393 (6)	−0.0165 (5)	0.0135 (5)	−0.0066 (5)
N1	0.0258 (7)	0.0239 (6)	0.0197 (6)	−0.0091 (5)	0.0022 (5)	−0.0034 (5)
N2	0.0252 (7)	0.0218 (6)	0.0180 (6)	−0.0073 (5)	0.0039 (5)	−0.0013 (5)
N3	0.0208 (6)	0.0217 (6)	0.0215 (6)	−0.0029 (5)	0.0001 (5)	−0.0020 (5)
C1	0.0296 (9)	0.0324 (8)	0.0274 (8)	−0.0096 (7)	0.0076 (6)	−0.0048 (7)
C2	0.0199 (7)	0.0257 (8)	0.0206 (7)	−0.0034 (6)	0.0013 (5)	0.0006 (6)
C3	0.0198 (7)	0.0216 (7)	0.0209 (7)	−0.0017 (5)	0.0010 (5)	0.0001 (6)
C4	0.0182 (7)	0.0199 (7)	0.0213 (7)	−0.0013 (5)	0.0014 (5)	0.0012 (6)
C5	0.0176 (7)	0.0215 (7)	0.0216 (7)	0.0001 (6)	0.0002 (5)	−0.0010 (6)
C6	0.0317 (9)	0.0284 (8)	0.0227 (7)	−0.0091 (7)	0.0055 (6)	−0.0051 (7)
C7	0.0497 (11)	0.0533 (11)	0.0422 (10)	−0.0325 (9)	0.0220 (8)	−0.0074 (9)
C8	0.0750 (15)	0.0473 (11)	0.0461 (11)	−0.0330 (10)	0.0243 (10)	−0.0123 (9)
C9	0.0187 (7)	0.0203 (7)	0.0181 (7)	−0.0021 (5)	0.0029 (5)	0.0003 (5)
C10	0.0188 (7)	0.0236 (7)	0.0214 (7)	0.0022 (6)	−0.0006 (5)	0.0007 (6)
C11	0.0247 (8)	0.0166 (7)	0.0244 (7)	0.0005 (6)	0.0021 (6)	−0.0005 (6)
C12	0.0188 (7)	0.0242 (7)	0.0159 (6)	−0.0029 (5)	0.0037 (5)	−0.0014 (5)
C13	0.0194 (7)	0.0250 (8)	0.0227 (7)	0.0014 (6)	−0.0028 (6)	0.0013 (6)
C14	0.0258 (8)	0.0170 (7)	0.0250 (7)	0.0014 (6)	0.0006 (6)	0.0016 (6)
C15	0.0295 (8)	0.0238 (8)	0.0229 (7)	−0.0064 (6)	0.0005 (6)	−0.0007 (6)
C16	0.0216 (8)	0.0375 (9)	0.0312 (8)	−0.0033 (6)	0.0012 (6)	−0.0079 (7)

### Geometric parameters (Å, °)

**Table:**

F2—C1	1.3403 (17)	C4—H4	0.9800
F3—C1	1.3353 (17)	C7—C8	1.472 (2)
F1—C1	1.3348 (18)	C7—H7A	0.9700
O1—C2	1.4149 (17)	C7—H7B	0.9700
O1—H1	0.860 (9)	C8—H8A	0.9600
O2—C5	1.2335 (16)	C8—H8B	0.9600
O3—C6	1.2119 (19)	C8—H8C	0.9600
O4—C6	1.3271 (19)	C9—C10	1.3828 (19)
O4—C7	1.4601 (18)	C9—C14	1.3938 (19)
N1—C5	1.3722 (18)	C10—C11	1.3878 (19)
N1—C2	1.4273 (17)	C10—H10	0.9300
N1—H1A	0.896 (9)	C11—C12	1.3956 (19)
N2—C5	1.3486 (18)	C11—H11	0.9300
N2—C4	1.4578 (17)	C12—C13	1.4021 (19)
N2—H2A	0.902 (9)	C13—C14	1.3805 (19)
N3—C12	1.4145 (17)	C13—H13	0.9300
N3—C15	1.4640 (17)	C14—H14	0.9300
N3—C16	1.4735 (18)	C15—H15A	0.9600
C1—C2	1.534 (2)	C15—H15B	0.9600
C2—C3	1.5397 (19)	C15—H15C	0.9600
C3—C6	1.5067 (19)	C16—H16A	0.9600
C3—C4	1.5429 (18)	C16—H16B	0.9600
C3—H3	0.9800	C16—H16C	0.9600
C4—C9	1.5079 (19)		
			
C2—O1—H1	104.5 (13)	O4—C7—H7A	109.8
C6—O4—C7	117.02 (14)	C8—C7—H7A	109.8
C5—N1—C2	124.51 (12)	O4—C7—H7B	109.8
C5—N1—H1A	114.4 (11)	C8—C7—H7B	109.8
C2—N1—H1A	119.6 (10)	H7A—C7—H7B	108.2
C5—N2—C4	122.66 (11)	C7—C8—H8A	109.5
C5—N2—H2A	118.1 (10)	C7—C8—H8B	109.5
C4—N2—H2A	115.7 (10)	H8A—C8—H8B	109.5
C12—N3—C15	115.84 (11)	C7—C8—H8C	109.5
C12—N3—C16	114.14 (11)	H8A—C8—H8C	109.5
C15—N3—C16	113.82 (11)	H8B—C8—H8C	109.5
F1—C1—F3	107.46 (12)	C10—C9—C14	118.10 (12)
F1—C1—F2	107.01 (12)	C10—C9—C4	120.13 (12)
F3—C1—F2	106.92 (12)	C14—C9—C4	121.75 (12)
F1—C1—C2	112.17 (12)	C9—C10—C11	121.39 (12)
F3—C1—C2	111.93 (12)	C9—C10—H10	119.3
F2—C1—C2	111.06 (12)	C11—C10—H10	119.3
O1—C2—N1	110.21 (11)	C10—C11—C12	120.54 (13)
O1—C2—C1	107.38 (11)	C10—C11—H11	119.7
N1—C2—C1	107.45 (12)	C12—C11—H11	119.7
O1—C2—C3	111.44 (11)	C11—C12—C13	117.92 (12)
N1—C2—C3	109.29 (11)	C11—C12—N3	122.47 (12)
C1—C2—C3	110.99 (11)	C13—C12—N3	119.60 (12)
C6—C3—C2	112.79 (11)	C14—C13—C12	120.75 (12)
C6—C3—C4	108.95 (11)	C14—C13—H13	119.6
C2—C3—C4	106.95 (11)	C12—C13—H13	119.6
C6—C3—H3	109.4	C13—C14—C9	121.09 (12)
C2—C3—H3	109.4	C13—C14—H14	119.5
C4—C3—H3	109.4	C9—C14—H14	119.5
N2—C4—C9	111.69 (11)	N3—C15—H15A	109.5
N2—C4—C3	106.48 (11)	N3—C15—H15B	109.5
C9—C4—C3	112.54 (11)	H15A—C15—H15B	109.5
N2—C4—H4	108.7	N3—C15—H15C	109.5
C9—C4—H4	108.7	H15A—C15—H15C	109.5
C3—C4—H4	108.7	H15B—C15—H15C	109.5
O2—C5—N2	121.65 (12)	N3—C16—H16A	109.5
O2—C5—N1	120.72 (12)	N3—C16—H16B	109.5
N2—C5—N1	117.58 (12)	H16A—C16—H16B	109.5
O3—C6—O4	125.44 (14)	N3—C16—H16C	109.5
O3—C6—C3	123.25 (14)	H16A—C16—H16C	109.5
O4—C6—C3	111.27 (13)	H16B—C16—H16C	109.5
O4—C7—C8	109.59 (14)		
			
C5—N1—C2—O1	93.48 (15)	C2—N1—C5—N2	7.3 (2)
C5—N1—C2—C1	−149.82 (13)	C7—O4—C6—O3	−5.4 (2)
C5—N1—C2—C3	−29.31 (18)	C7—O4—C6—C3	172.21 (12)
F1—C1—C2—O1	−179.00 (11)	C2—C3—C6—O3	−53.65 (18)
F3—C1—C2—O1	60.12 (15)	C4—C3—C6—O3	64.94 (17)
F2—C1—C2—O1	−59.30 (14)	C2—C3—C6—O4	128.71 (12)
F1—C1—C2—N1	62.46 (15)	C4—C3—C6—O4	−112.70 (13)
F3—C1—C2—N1	−58.43 (15)	C6—O4—C7—C8	−94.93 (19)
F2—C1—C2—N1	−177.85 (11)	N2—C4—C9—C10	128.63 (13)
F1—C1—C2—C3	−56.98 (16)	C3—C4—C9—C10	−111.65 (14)
F3—C1—C2—C3	−177.86 (11)	N2—C4—C9—C14	−52.72 (16)
F2—C1—C2—C3	62.72 (15)	C3—C4—C9—C14	67.00 (15)
O1—C2—C3—C6	52.81 (15)	C14—C9—C10—C11	−3.27 (19)
N1—C2—C3—C6	174.86 (11)	C4—C9—C10—C11	175.43 (12)
C1—C2—C3—C6	−66.81 (15)	C9—C10—C11—C12	−0.2 (2)
O1—C2—C3—C4	−66.94 (13)	C10—C11—C12—C13	4.00 (19)
N1—C2—C3—C4	55.11 (14)	C10—C11—C12—N3	−177.11 (11)
C1—C2—C3—C4	173.44 (11)	C15—N3—C12—C11	6.82 (18)
C5—N2—C4—C9	165.79 (12)	C16—N3—C12—C11	141.96 (13)
C5—N2—C4—C3	42.56 (16)	C15—N3—C12—C13	−174.31 (12)
C6—C3—C4—N2	177.15 (11)	C16—N3—C12—C13	−39.17 (16)
C2—C3—C4—N2	−60.65 (13)	C11—C12—C13—C14	−4.37 (19)
C6—C3—C4—C9	54.46 (15)	N3—C12—C13—C14	176.70 (12)
C2—C3—C4—C9	176.66 (11)	C12—C13—C14—C9	1.0 (2)
C4—N2—C5—O2	168.05 (12)	C10—C9—C14—C13	2.89 (19)
C4—N2—C5—N1	−14.73 (19)	C4—C9—C14—C13	−175.79 (12)
C2—N1—C5—O2	−175.47 (12)		

### Hydrogen-bond geometry (Å, °)

**Table:**

*D*—H···*A*	*D*—H	H···*A*	*D*···*A*	*D*—H···*A*
O1—H1···O3	0.86 (1)	1.97 (1)	2.7601 (16)	153 (2)
N1—H1A···O2^i^	0.90 (1)	1.91 (1)	2.8049 (15)	174 (2)
N2—H2A···N3^ii^	0.90 (1)	2.12 (1)	3.0241 (18)	178 (2)

Symmetry codes: (i) −*x*+2, −*y*+2, −*z*+2; (ii) −*x*+3/2, *y*+1/2, −*z*+3/2.
